# Preoperative dynamic quantitative sensory testing in remote pain-free areas is associated with axial pain after posterior cervical spinal surgeries

**DOI:** 10.1186/s12891-022-05366-x

**Published:** 2022-05-02

**Authors:** Kaiwen Chen, Jie Yu, Cong Nie, Yu Zhu, Jianyuan Jiang, Wei Lei, Xinlei Xia, Chaojun Zheng

**Affiliations:** 1 Department of Orthopedics, Huashan Hospital, Fudan University, 12 Mid- Wulumuqi Road, Shanghai, 200040 China; 2 Department of Infectious Diseases, Huashan Hospital, Fudan University, Shanghai, 200040 China; 3 Department of Physical Medicine and Rehabilitation, Upstate Medical University, State University of New York at Syracuse, Syracuse, NY 10212 USA; 4 Department of Nursing, Huashan Hospital, Fudan University, Shanghai, 200040 China

**Keywords:** Postoperative axial pain, Quantitative sensory testing, Temporal summation, Conditioned pain modulation, Posterior cervical spinal surgery

## Abstract

**Background:**

Postoperative axial pain (PAP), characterized by pain and/or stiffness around the posterior neck, periscapular areas and/or shoulder region, is a vexing complication affecting 5–60% of patients undergoing posterior cervical decompression. Given its relatively high frequency and negative impact on patients’ physical and mental status, efforts preoperatively to confirm patients at risk of developing PAP to offer more efficient pain management to minimize this complication have a high priority. The aim of this study is to investigate the role of preoperative dynamic quantitative sensory testing (QST) in predicting the PAP after posterior cervical decompression.

**Methods:**

This longitudinal observational study included 122 patients with degenerative cervical myelopathy undergoing laminoplasty or laminectomy. Preoperatively, all patients underwent the assessment of pressure pain thresholds (PPTs) at local and remote pain-free areas and both temporal summation (TS) and conditioned pain modulation (CPM) at remote pain free-areas. These patients underwent further pain-related, psychosocial and clinical function assessments before and/or after operation.

**Results:**

In the present study, 21 patients (21/122, 17.2%) developed PAP, and the 6-month postoperative follow-up demonstrated that 8 of these 21 patients developed chronic PAP (CPAP). All preoperative covariates with significant differences between the PAP and non-PAP groups were subjected to multivariate logistic regression, and the presence of preoperative axial pain, surgical plan including C2 decompression, total international physical activity questionnaire score (cutoff value [CV]: 2205.5, sensitivity: 82.4%; specificity: 61.1%) and TS value (CV: 2.5, sensitivity: 42.9%; specificity: 83.2%) were independently associated with PAP (*P* < 0.05). Logistic regression further revealed that the presence of preoperative axial pain, TS value (CV: 2.5, sensitivity: 62.5%; specificity: 83.2%) and CPM value (CV: 0.65, sensitivity: 87.5%; specificity: 61.4%) were significant predictors of CPAP (*P* < 0.05).

**Conclusions:**

The findings of this study support the hypothesis that preoperative endogenous pain modulation efficiency may be associated with axial pain after posterior cervical decompression. Clinically, preoperative estimation of both TS and CPM in remote pain-free areas may provide additional useful information for identifying patients who may be at risk of developing both PAP and CPAP, which may be beneficial in enabling stratification in the perioperative period of patients based on individual vulnerabilities to avoid/reduce this complication.

**Supplementary Information:**

The online version contains supplementary material available at 10.1186/s12891-022-05366-x.

## Background

Posterior cervical decompression has been widely used to treat degenerative cervical myelopathy (DCM). However, serious complications can occur [1–6]. Among these, postoperative axial pain (PAP), characterized by pain and/or stiffness around the posterior neck, periscapular areas and/or shoulder region, is a vexing complication affecting 5–60% of patients undergoing posterior cervical decompression [4–6]. Some previous studies further suggested that this annoying pain may persist for several years after surgery [6, 7]. Importantly, it is evident that the treatments of chronic postoperative pain are difficult because of the limited amount of available efficient drugs and the undesired side effects [8]. Therefore, efforts to explore reliable methods to preoperatively identifying patients who may be at risk of developing PAP and/or chronic PAP (CPAP) to offer personalised care or personalised medicine (e.g., reducing muscle detachment, reconstructing muscle insertion, changing surgical approach and using higher doses analgesics) [5, 8, 9] to minimize this complication have a high priority.

Increasing studies has indicated that the preoperative pain response to noxious stimulation is associated with acute/chronic pain after many different kinds of surgeries [10–12]. Furthermore, a systematic review included 14 studies investigating the correlation between preoperative responses to experimental pain stimuli and postoperative pain demonstrated that the preoperative pain tests may predict 4–54% of the variance in postoperative pain experience depending on the stimulation methods and the test paradigm used [13]. All of these evidences suggested that endogenous pain systems may be involved in the pathophysiological process of postoperative pain. Kimura et al. and Yoshida et al. demonstrated that pre-existing pain and poor mental health status before operation are significant risk factors for PAP [5, 6], and it is generally accepted that both features are closely associated with endogenous pain modulation efficiency [10, 12, 14]. Thus, preoperative effectiveness of endogenous analgesia may be closely related to the formation of PAP. According to previous studies [11, 12, 15, 16], the mechanisms of endogenous analgesia involve pain facilitation and inhibition, which may be quantified by temporal summation (TS) and conditioned pain modulation (CPM). Both TS and CPM present the methods of dynamic quantitative sensory testing (QST) and reflect function of central facilitation and modulation of incoming nociceptive signals [17]. Recently published systematic review demonstrates that TS and CPM show the most consistent predictive values for chronic postoperative pain, especially in the orthopedic-related surgeries [8]. As such, preoperative investigation of these QST to evaluate preoperative endogenous analgesia may provide additional information for predicting PAP.

Hence, the aim of the present study was to investigate these dynamic QST measurements in patients with DCM before posterior cervical decompression, and the role of these dynamic QST techniques in predicting PAP after posterior cervical spinal surgeries was also analyzed.

## Methods

All methods used in this longitudinal observational study were carried out in accordance with ‘Declaration of Helsinki’, and the study protocol was approved by Human Ethics Committees (Huashan Hospital, Fudan University, China). All subjects gave informed consent through written.

## Subjects

This study included the patients with cervical spondylotic myelopathy (CSM) or ossification of the posterior longitudinal ligament (OPLL) who underwent posterior cervical decompression (from January 2017 to September 2020 in Huashan Hospital).

The inclusion criteria included a confirmed diagnosis of multilevel cervical pathologies including CSM or OPLL identified by clinical symptoms, magnetic resonance imaging (MRI), electrophysiological detection, and operative findings. The exclusion criteria included previous spinal surgery, cervical structural pathologies (e.g., trauma, infections and tumor), coexisting conditions that could increase procedural risk or worsen/change the pain (e.g., severe hypertension or cardiac disease, peripheral vascular disease, shoulder impairment, and neurological disease).

### Anesthesia management, surgical treatment and perioperative analgesia

Total intravenous anesthesia (TIVA) was used in all patients in this study because of intraoperative monitoring. Anesthesia was induced with a propofol infusion combined with a single bolus of non-depolarizing short-acting muscle relaxant (vecuronium or atracurium). Anesthesia was maintained with a propofol infusion (6–10 mg/kg/h) and remifentanil (0.15–0.35 μg/kg/min).

**Fig. 1:**
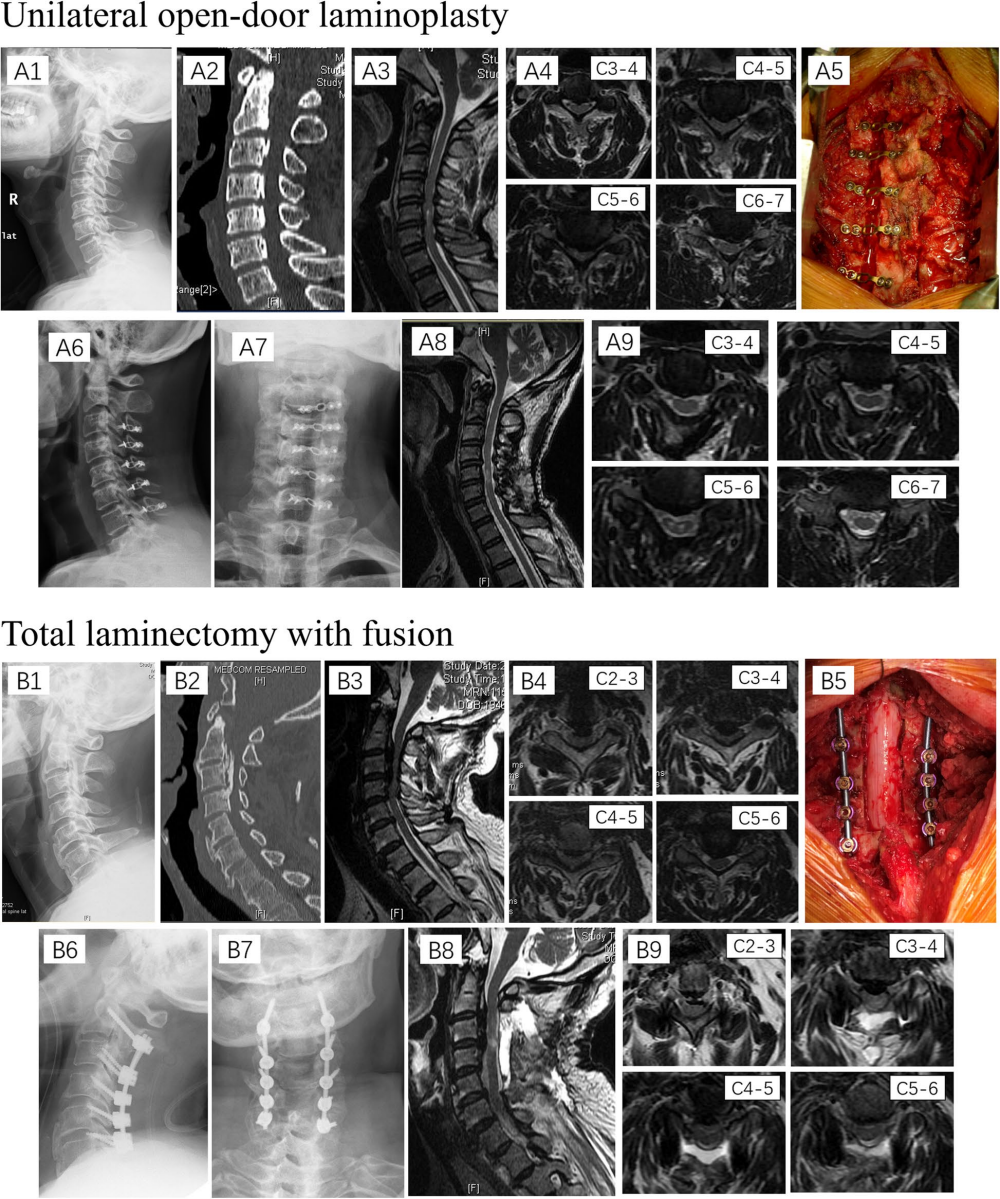
Imaging illustrations of both unilateral open-door laminoplasty and total laminectomy with fusion. **A1–4** Preoperative imaging revealed multi-segmental cervical canal stenosis due to OPLL and thickening of the ligamentum flavum; **A5** Intraoperative view of left-side open-door laminoplasty at C3–7 with plate fixation; **A6–7** Postoperative imaging showed left-side open-door laminoplasty at C3–7 with plate fixation; **A8–9** Postoperative MRI showed obviously reduced cervical cord compression after unilateral open-door laminoplasty. **B1–4** Preoperative imaging revealed multi-segmental cervical canal stenosis due to OPLL; **B5** Intraoperative view of total laminectomy with fusion and internal fixation; **B6–7** Postoperative imaging showed total laminectomy with fusion and fusion and internal fixation from segments C3 to C7; **B8–9** Postoperative MRI showed obviously reduced cervical cord compression after total laminectomy; OPLL: ossification of the posterior longitudinal ligament; MRI: magnetic resonance imaging

All patients underwent unilateral open-door laminoplasty or total laminectomy with fusion (Fig. 1), and the operative segments were decided based on both clinical examination and imaging findings. At the end of the operation, all detached paraspinal muscles were resutured with the original spinous process if the spinous processes were persevered. All surgeries in this study were performed by the same team, and the operating surgeons of this team had more than 10 years of experience in spinal surgeries.

Preoperatively, the patients usually did not take analgesic drugs, and only 6 patients with preoperative pain in this study were treated with acetaminophen every 12 hours, and all of these 6 patients stopped the analgesic drugs 1 day before operation. Postoperatively, local infiltration around the incision using 20 ml 0.75% ropivacaine was performed. Furthermore, a flurbiprofen axetil injection (50 mg) was given during the first 24 hours postoperatively. Afterwards, patients were instructed to take acetaminophen (450 mg) every 12 hours during the first 5 days postoperatively. If the patient had moderate or severe pain, supplemented (e.g., ibuprofen, celecoxib and opioids) and/or prolonged use (e.g., flurbiprofen axetil injection) of pain medication was allowed. In addition, patients were usually discharged from the hospital 5–10 days after operation, and the cervical spine was immobilized with a neck-collar for approximately 3 weeks.

## Clinical assessments

### Quantitative sensory testing

Both TS and CPM, as well as pressure pain threshold (PPT), were performed in all patients 1–3 days before operation using a pressure algometer (FDN 100, Wagner Instruments, Greenwich, CT, USA). The procedures of QST were as follows: (1) All patients were asked to refrain from using analgesics for treating pain and consuming caffeine, alcohol, or nicotine 1 day before the assessment; (2) Before the QST assessments, the procedures were introduced to all subjects in detail until they were familiar with these methods; (3) First, PPTs were recorded from both local and remote pain-free areas; (4) After PPT recordings, TS were recorded from the remote pain-free area; (5) At last, CPM were recorded from the remote pain-free area.

**Fig. 2:**
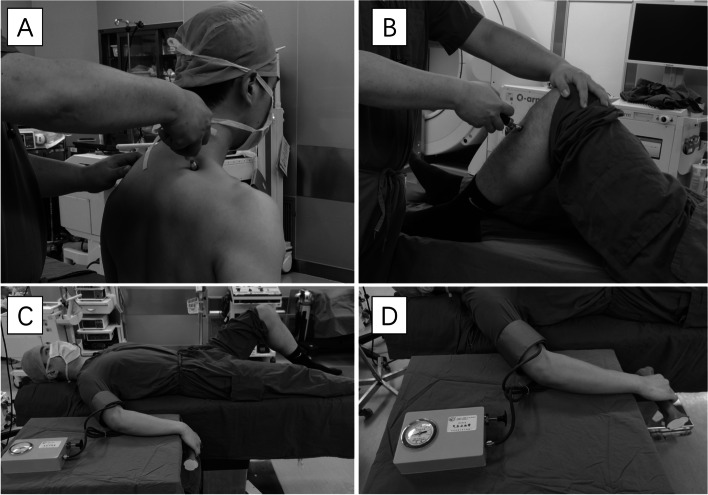
Illustrations of QST assessments. **A** PPT tests at the upper fibers of the trapezius; **B** Both PPT and TS tests at the belly of the tibialis anterior; **C-D** Conditioning painful stimulus of the CPM at the upper arm. QST: quantitative sensory testing; PPT: pressure pain threshold; TS: temporal summation; CPM: conditioned pain modulation

During the PPT assessments, the measurements were recorded from two different regions in each subject (Fig. 2): (1) upper fibers of the trapezius on the side with more pain or on the right side for pain-free cases (local area), and (2) belly of the tibialis anterior on the left side (remote pain-free area). The PPT was assessed three times in each region with an interstimulus interval of at least 60 seconds, and the average of these 3 assessments was recorded as PPT for this region. If the subjects failed to report pain to a pressure of 10 kg/cm^2^, the test was stopped, and this value was recorded as PPT.

During the TS assessments, 10 sequential mechanical pressure stimulations with the algometer at the pain threshold level (at 1-s intervals with the assistance of a metronome) were performed in the remote pain-free area (Fig. 2) [18, 19]. The subjects were asked to rate their pain with the 1st and 10th pulses on a numeric rating scale (NRS), and the rating of TS resulted from the difference between these two NRS scores. Compared with using other sharp objects (e.g., a pin-prick, nylon filament), using pressure algometry to evaluate TS avoids measurement error associated with the uneven application of force over repeated measures.

During the CPM assessments, conditioning stimulus was performed by applying intense pressure to the right upper arm with an 11-cm wide tourniquet to temporarily induce ischemic pain (Fig. 2) [20, 21]. The pressure cuff was inflated to approximately 260 mmHg, and the subjects were asked to lift a dumbbell (1 kg for females and 2 kg for males) by stretching their wrist until they completed 45 wrist lifts or when the pain intensity of upper arm reached 7 or more on NRS (Fig. 2) since a previous study demonstrated that the CPM effect increases with increased conditioning stimulus intensity [22]. During the conditioning painful stimulus, parallel pain threshold (test stimulus) was repeated in remote pain-free areas, and the differences between the PPT with and without the conditioning stimulation was recorded as CPM.

## Questionnaires

All patients were asked to rate the axial pain intensity at rest before operation using the NRS, and the average neck pain intensity 1–5 days after operation was also recorded. Furthermore, all patients were routinely followed-up at 1 month, 6 months and 12 months after operation by telephone or in the clinic, and pain intensity was evaluated in these patients with PAP. In this study, PAP was defined as newly developed or worsening pain (greater than or equal to 3 on the NRS) around the neck and shoulder persisting for at least 1 month [4, 23], and persistent or recurrent PAP more than 6 months after operation was considered CPAP.

Preoperatively, all patients further accepted multiple questionnaires, including the Pain Catastrophizing Scale (PCS), Beck Anxiety Inventory (BAI), Beck Depression Index (BDI) and International Physical Activity Questionnaire (IPAQ), and all of these questionnaires have been validated in the Chinese environment in the previous studies [24–27]. Cervical Japanese Orthopedic Association (JOA) scores were evaluated before and 1 year after operation, and the postoperative one-year neurological recovery rate was measured.

## Statistical methods

All data were analyzed using SPSS version 20.0 (IBM, Armonk, NY), and the Kolmogorov-Smirnov test was used to confirm a normal distribution. Pearson or Spearman correlation analysis was used to evaluate the relationship between the PAP/CPAP intensity and perioperative measurements. The measurements for the non-PAP and PAP/CPAP patient groups were compared using independent t-tests or Mann-Whitney tests, and the frequencies of the different measurements between the patients without PAP and those with PAP/CPAP were compared by Fisher’s exact test or chi-square test. Statistically significant preoperative covariates based on univariate analyses were subjected to multivariate logistic regression analysis, and the cutoff values (CV) and area under the curve (AUC) of the results of the multivariate logistic regression were identified by a receiver operating characteristic (ROC) curve. A *P*-value < 0.05 was considered significant.

## Results

This study included 122 patient subjects (male vs. female: 96 vs. 34; age: 57.9 ± 11.0 years), and the medical characteristics of these patients were listed in Table 1.

**Table 1:** Demographic and medical characteristics between patients with and without PAP

	Patients without PAP	Patients with PAP	Patients with CPAP^^^
**Number of subjects**	101	21	8
**Age range (years)**	58.5 ± 11.6	55.3 ± 7.0	52.8 ± 5.5^#^
**BMI**	24.1 ± 2.6	24.9 ± 2.9	25.6 ± 3.0
**Gender (Male vs. Female)**	79 vs. 22	14 vs. 7	3 vs. 5^#^
**Disease duration (months)**	14.1 ± 19.2	18.3 ± 19.9	21.4 ± 22.9
**Preoperative radiographic measurement**			
Cervical curvature (°)	13.3 ± 10.9	13.3 ± 11.6	15.1 ± 13.2
C7 slope (°)	24.4 ± 7.9	22.8 ± 8.6	21.7 ± 9.9
C2 SVA (mm)	19.1 ± 16.1	20.7 ± 16.0	22.0 ± 13.9
**Main diagnosis (n/total cases (%))**			
CSM	68/101 (67.3%)	10/21 (47.6%)	4/8 (50.0%)
Cervical OPLL	33/101 (32.7%)	11/21 (52.4%)	4/8 (50.0%)
**Surgical information:**			
Operative time (min)	119.3 ± 20.0	127.7 ± 33.1	130.9 ± 35.9
Intraoperative bleeding (ml)	266.3 ± 72.7	290.0 ± 73.4	314.9 ± 75.4
Number of surgical segments	4.8 ± 0.6	4.9 ± 0.9	5.0 ± 1.1
Laminoplasty (n/total cases (%))	88/101 (87.1%)	16/21 (76.2%)	6/8 (62.5%)
Laminectomy (n/total cases (%))	13/101 (12.9%)	5/21 (23.8%)	2/8 (27.5%)
Surgery involves C2 (n/total cases (%))	7/101 (6.9%)	8/21 (38.1%)^*^	3/8 (37.5%)^#^
Surgery involves C7 (n/total cases (%))	69/101 (68.3%)	12/21 (57.1%)	5/8 (62.5%)
**Complication (n/total cases (%))**			
Dura defect	4/101 (4.0%)	1/21 (4.8%)	1/8(12.5%)
Surgical site infection	5/101 (5.0%)	2/21 (9.5%)	0/8(0.0%)
Nerve damage	5/101 (5.0%)	2/21 (9.5%)	1/8 (12.5%)

Measurements are expressed as the mean ± SD

*SVA* Sagittal vertical axis, *CSM* Cervical spondylotic myelopathy, *OPLL* Ossification of the posterior longitudinal ligament, *PAP* Postoperative axial pain, *CPAP* Chronic postoperative axial pain, *BMI* Body mass index

^*^Statistically significant differences between the non-PAP and PAP patient groups

^#^Statistically significant differences between the non-PAP and CPAP patient groups

^^^Eight of 21 patients with PAP developed CPAP; thus, the measurements of patients with CPAP were included in the measurements of those with PAP

In the present study, 21 patients (21/122, 17.2%) presented with PAP after posterior cervical surgeries, and the postoperative 1-month follow-up NRS score in these patients was 3.8 ± 0.9. In these patients, 16 (16/21, 76.2%) patients used analgetic medication, including Acetaminophen (*n* = 7), Codeine (*n* = 1), Tramadol (*n* = 1), Ibuprofen (*n* = 2) and Celecoxib (*n* = 5).

The 6-month postoperative follow-up demonstrated that 8 of these 21 patients (8/122, 6.6%) developed CPAP (NRS: 3.9 ± 0.8), and 6 of these 8 patients still had axial pain at the last follow-up (NRS: 3.5 ± 0.5).

### Correlations between the PAP/CPAP intensity and perioperative measurements

In the patients with PAP, PAP intensity was correlated with moderate IPAQ (*r* = − 0.54, *P* < 0.05), PCS (*r* = 0.45, *P* < 0.05), TS (*r* = 0.53, *P* < 0.05) and average pain intensity 1–5 days postoperatively (*r* = 0.56, *P* < 0.05). In the CPAP patient group, there was a significant correlation between CPAP intensity and average pain intensity 1–5 days postoperatively (*r* = 0.74, *P* < 0.05) and CPM (*r* = − 0.78, *P* < 0.05).

### Comparison between the patients with and without PAP/CPAP

In the current study, the patients with PAP showed a higher proportion of cases accepting C2 decompression, a higher proportion of cases with preoperative axial pain, higher PCS score, lower IPAQ score, lower PPT in the local area, lower CPM and greater TS than those in patients without PAP (Tables 1, 2 and 3).

**Table 2:** Clinical scores between patients with and without PAP/CPAP

	Non-PAP	PAP	CPAP^^^
**Number of subjects**	101	21	8
**Pain assessment**			
Preoperative NRS	2.3 ± 0.5 (23/101)	2.6 ± 0.7 (12/21^*^)	2.8 ± 0.8 (5/8^#^)
Postoperative average NRS^$^	2.8 ± 0.6	3.0 ± 0.5	3.3 ± 0.6^#^
**Cervical cord function assessment**			
Preoperative JOA	10.1 ± 3.4	9.4 ± 3.0	9.3 ± 1.6
Postoperative JOA	13.8 ± 2.3	13.1 ± 2.0	12.5 ± 1.7
JOA improvement rates (%)	55.7 ± 15.1	50.1 ± 12.8	43.6 ± 13.1^#^
**Preoperative psychological assessment (n/total cases (%))**			
PCS scores	9.7 ± 6.3	12.6 ± 5.5^*^	15.1 ± 5.8^#^
BAI scores	5.7 ± 4.9	7.3 ± 5.3	9.3 ± 5.8
*Mild anxiety*	7/101 (6.9%)	2/21 (9.5%)	1/8 (12.5%)
*Moderate anxiety*	/	/	/
*Severe anxiety*	/	/	/
BDI scores	4.3 ± 4.2	5.9 ± 4.3	8.1 ± 4.2^#^
*Mild emotional distress*	12/101 (11.9%)	4/21 (19.0%)	3/8 (37.5%)^#^
*Marginal depression*	/	/	/
*Moderate depression*	/	/	/
*Severe depression*	/	/	/
*Extremely severe depression*	/	/	/
**Preoperative physical activity assessment**			
Total IPAQ^&^	2314.6 ± 459.0 (90)	1999.9 ± 292.1^*^ (17)	1884.9 ± 95.2^#^ (7)
Vigorous IPAQ	692.4 ± 306.4	509.4 ± 244.3^*^	525.7 ± 173.5^#^
Moderate IPAQ	832.7 ± 233.4	692.0 ± 169.2^*^	594.6 ± 186.5^#^
Walking	789.4 ± 186.9	798.5 ± 183.5	764.6 ± 138.7

*PAP* Postoperative axial pain, *CPAP* Chronic PAP, *BMI* Body Mass Index, *NRS* Numeric Rating Pain Scale, *JOA* Japanese Orthopaedic Association, *PCS* Pain Catastrophizing Scale, *BAI* Beck Anxiety Inventory, *BDI* Beck Depression Index, *IPAQ* International Physical Activity Questionnaire

^(a/b)^a is the number of patients with preoperative axial pain, and b is the total number of patients

^(c)^c is the number of patients who accepted the IPAQ assessment

^*^Statistically significant differences between the non-PAP and PAP patient groups

^#^Statistically significant differences between the non-PAP and CPAP patient groups

^^^Eight of 21 patients with PAP developed CPAP; thus, the measurements of patients with CPAP were included in the measurements of those with PAP

^$^Average pain intensity 1–5 days after operation

^/^no patient

^&^Total IPAQ scores = Vigorous IPAQ + Moderate IPAQ +Walking

**Table 3:** QST measurements between patients with and without PAP

	Patient without PAP	Patients with PAP	Patients with CPAP^
**Number of subjects**	101	21	8
PPT in neck (Kg/cm^2^)	4.5 ± 2.1	3.5 ± 1.7*	3.1 ± 1.4#
PPT in TA (Kg/cm^2^)	3.7 ± 1.4	3.4 ± 1.4	3.1 ± 1.2
CPM (Kg/cm^2^)	0.8 ± 0.6	0.5 ± 0.6*	0.2 ± 0.6#
TS	1.6 ± 1.0	2.4 ± 1.1*	2.8 ± 1.0#

Measurements are expressed as the mean ± SD

*QST* Quantitative sensory testing, *PAP* Postoperative axial pain, *CPAP* Chronic postoperative axial pain, *PPT* Pressure pain threshold, *CPM* Conditioned pain modulation, *TS* Temporal summation

^*^Statistically significant differences between patients with and without PAP

^#^Statistically significant differences between patients without PAP and patients with CPAP

^^^Eight of 21 patients with PAP developed CPAP; thus, all measurements of patients with CPAP were included in the measurements of those with PAP

Compared with non-PAP group, the patients with CPAP had a younger age, higher proportion of females, higher proportion of cases accepting C2 decompression, higher proportion of cases with preoperative axial pain, higher PCS score, higher BDI score, lower IPAQ score, lower PPT in the local area, lower CPM and greater TS (Tables 1, 2 and 3). These CPAP patients also presented with a higher average NRS score 1–5 days after operation and lower JOA improvement rates 1 year after operation (Tables 1 and 2).

### Preoperative predictors of PAP/CPAP after posterior cervical surgeries

**Fig. 3:**
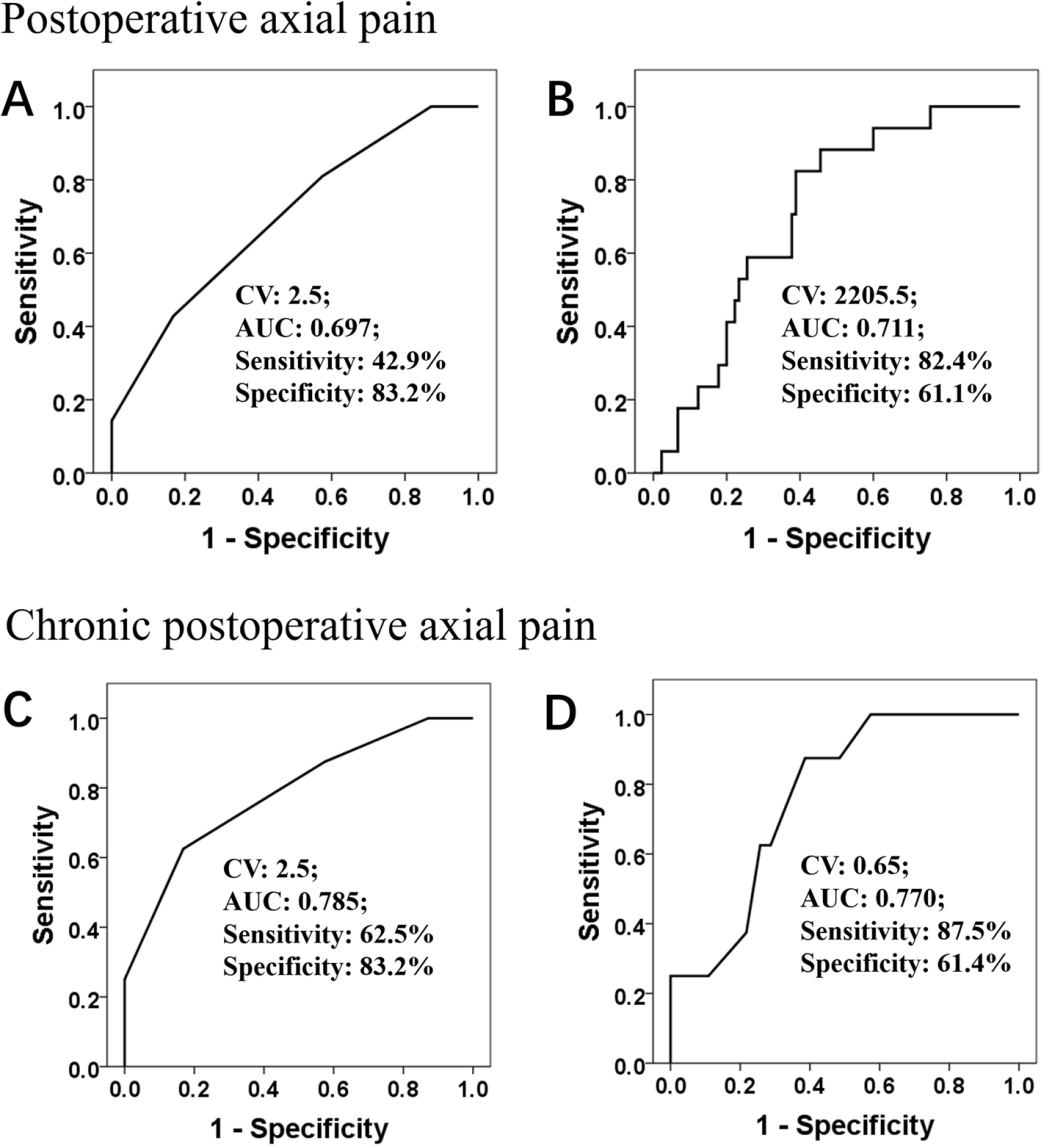
ROC curve analysis revealed the cutoff value of TS (**A**) and total IPAQ score (**B**) for predicting PAP after posterior cervical spinal surgeries, and ROC curve analysis revealed the cutoff value of TS (**C**) and CPM (**D**) for predicting chronic PAP after posterior cervical spinal surgeries. ROC curve: receiver operating characteristic curve; AUC: area under the curve; CV: cutoff value; PAP: postoperative axial pain; IPAQ: international physical activity questionnaire; TS: temporal summation; CPM: conditioned pain modulation

All preoperative covariates with significant differences (*P* < 0.05) between the PAP and non-PAP groups, including the presence of preoperative axial pain, surgical plan including C2 decompression, IPAQ, PCS, local-area PPT, CPM and TS, were subjected to multivariate logistic regression. The results showed that the cases with preoperative axial pain, surgical plan including C2 decompression, total IPAQ score, and TS were independently associated with PAP (Table 4). ROC curve analysis revealed the CV of TS (CV: 2.5) and total IPAQ (CV: 2205.5) for predicting PAP after posterior cervical surgeries (Fig. 3).

**Table 4:** Multivariate regression analysis of significant predicting factors of postoperative axial pain

	B	OR	CI	P
**Postoperative axial pain**				
C2 decompression	2.432	11.383	2.098–61.752	0.005
Preoperative axial pain	1.651	5.213	1.414–19.215	0.013
Total IPAQ score	−0.001	0.999	0.998–1.000	0.016
TS	0.848	2.335	1.151–4.737	0.019
**Chronic postoperative axial pain**				
Preoperative axial pain	2.057	7.826	1.030–59.455	0.047
CPM (Kg/cm^2^)	−2.366	0.094	0.010–0.879	0.038
TS	1.292	3.639	1.069–12.391	0.039

*CPM* Conditioned pain modulation, *TS* Temporal summation, *IPAQ* International Physical Activity Questionnaire, *CI* Confidence interval, *OR* Odds ratio, *P* P-value

All statistically significant preoperative measurements (*P* < 0.05) between the CPAP and non-PAP groups were subjected to multivariate logistic regression analysis, and the results revealed that the presence of preoperative axial pain, TS and CPM were significant predictors of CPAP. Furthermore, ROC curve analysis revealed the CV of CPM (CV: 0.65) and TS (CV: 2.5) for predicting CPAP (Fig. 3).

## Discussion

The significant incidence of PAP in this study indicates that approximately one in five patients undergoing posterior cervical decompression will have PAP. Importantly, almost one-third of these patients with PAP will develop chronic pain. Furthermore, similar to previous studies [5, 6], a significant difference in postoperative functional recovery was observed between the CPAP and non-PAP patient groups in this study. Therefore, it is worth clinicians being cautious about this clinically significant complication when they consider using posterior cervical surgeries to treat patients with DCM.

One of the key findings of this study is that both TS and CPM evaluated in remote pain-free areas before operation were associated with CPAP after posterior cervical surgeries. It is generally accepted that noxious stimuli can reduce the activity of dorsal horn nociceptive neurons and result in an increased pain threshold and decreased pain [28]. Thus, the CPM is considered to be a feasible method to evaluate pain inhibition. Recently published systematic review demonstrated that dynamic QST, including CPM, show the most consistent predictive values for chronic postoperative pain and analgesic effect, but the heterogeneous methodologies reduce the generalizability [8]. Furthermore, individual pain facilitating properties quantified by TS are another essential component of pain modulation, and previous studies have demonstrated that enhanced pain facilitation can amplify synaptic inputs of injury and/or expand the pain receptive field, generating an augmented action potential output and resulting in hyperalgesia and increased pain [29, 30]. Therefore, despite similar degrees of nociceptive stimuli, individual differences in the endogenous pain modulation efficiency may result in completely different pain experiences.

Importantly, preoperative-assessed TS but not CPM was associated with the axial pain at the early postoperative phase, which may be ascribed to different pathways of pain modulation reflected by TS and CPM. Based on rat and mouse models of traumatic brain injury, Irvine et al. demonstrated that nociceptive pain sensitization may mainly consist of two stages [31, 32]. During the early stage of nociceptive stimulation, it is mainly manifested in the enhancement of pronociceptive serotonergic signaling, resulting in an imbalance of endogenous pain modulation that favors descending pain facilitation [32], and descending pain inhibition may mainly play a role in the late stage of nociceptive stimulation [33]. In this study, PAP intensity was significantly correlated with TS, while CPAP strength was obviously related to CPM, which further supported this possibility. Increasing evidence demonstrates that pain experience during the postoperative acute stage increases the incidence of chronic pain [14, 34], and acute pain has been demonstrated to be a better target for pharmacological intervention than chronic pain [34]. Therefore, preoperative detection of TS in remote pain-free areas may have more important clinical significance.

In keeping with the results of previous studies on postoperative pain [12, 14], preoperative axial pain at rest was associated with PAP in this study. In contrast to pain with movement, pain at rest has been linked to central sensitization [14]. Therefore, preoperative central hypersensitivity caused by impaired endogenous pain regulation may be one of the possible risk factors for PAP/CPAP after posterior cervical decompression, which was further supported by many previous studies [14, 34]. Importantly, this hypothesis also provides a possible explanation for the predictive value of total IPAQ scores associated with PAP. The IPAQ is a subjective measure of the amount of time of physical activities during the past 7 days before assessment [35], and the significant difference in both vigorous and moderate IPAQ scores between the PAP and non-PAP patient groups in this study suggested that non-PAP patients may exercise more often. Recent accumulating studies have demonstrated that exercises can not only improve pain through exercise-induced analgesia [36, 37], but also reduce central sensitization associated with pain [36]. Coupled with the negative correlation between the moderate IPAQ and PAP intensity, we speculated that exercise may reduce both the intensity and incidence of PAP by improving the effectiveness of endogenous analgesia to alleviate preoperative central sensitization. This finding provides a possible method to prevent PAP, but more definite results need further prospective studies.

The findings of this study should be interpreted with caution since the main mechanism leading to PAP remains unclear. The current study demonstrated that the efficiency of preoperative endogenous pain modulatory systems may be associated with PAP/CPAP. However, laminoplasty/laminectomy at the C2 segments was also demonstrated to be a meaningful variable in predicting PAP in this study, suggesting that there were other pathogenic mechanisms (e.g., surgical trauma) related to PAP. According to the Table 2, greater number of CPAP patients had mild emotional distress relative to those without PAP. According to the previous study [30], there is bidirectional relationship between the central pain modulation and the psychosocial symptom; thus, the psychosocial symptom may also contribute to the CPAP. Furthermore, although it did not reach statistical significance, large difference between the incidence of operative complications, especially nerve damages, between the patients with and without PAP/CPAP suggested that nerve damages might also have an important effect on developing PAP or its chronicity. Hosono et al. speculated that intraoperative nerve root irritation caused by tip of drill may be possible reason for PAP [4], and Zhang et al. indicated that PAP may be ascribed to the iatrogenic injury to the medial branches ramified from the dorsal ramus of the cervical nerves along the dorsolateral part of the facet joints [38]. Therefore, further study is required to identify the different pathogenic mechanisms and their roles in PAP. Furthermore, the small sample size, especially the CPAP patient group, is a strong limitation of this study. However, significantly poorer postoperative functional recovery was observed in the patients with CPAP than in those without PAP, which is of great significance for both patients and clinicians. Therefore, the preliminary findings involving CPAP should be mentioned in this study, but more significant results might be achieved in a study with more cases.

## Conclusions

The findings of this study demonstrated that preoperative endogenous pain modulation efficiency may be involved in the pathophysiological process of axial pain after posterior cervical decompression. Preoperative estimation of dynamic QST, including both TS and CPM, in remote pain-free areas may provide additional useful information for identifying patients who may be at risk of developing both PAP and CPAP after posterior cervical spinal surgeries, which may be beneficial in enabling stratification in the perioperative period of patients based on individual vulnerabilities to avoid/reduce this complication.

## Supplementary Information

**Additional file 1.**

## Data Availability

The datasets have been presented in the main manuscript, and the raw data used and/or analysed during the current study are available in the supplementary file 1.

## Abbreviations

DCM: Degenerative cervical myelopathy
PAP: Postoperative axial pain
PPTs: Pressure pain thresholds
TS: Temporal summation
CPM: Conditioned pain modulation
QST: Quantitative sensory testing
CSM: Cervical spondylotic myelopathy
OPLL: Ossification of the posterior longitudinal ligament
NRS: Numeric rating scale
CPAP: Chronic postoperative axial pain
PCS: Pain Catastrophizing Scale
BAI: Beck Anxiety Inventory
BDI: Beck Depression Index
IPAQ: International Physical Activity Questionnaire
JOA: Japanese Orthopedic Association
AUC: Area under the curve
ROC: Receiver operating characteristic
